# Solid Polymer Electrolytes with High Conductivity and Transference Number of Li Ions for Li‐Based Rechargeable Batteries

**DOI:** 10.1002/advs.202003675

**Published:** 2021-02-08

**Authors:** Yun Zhao, Li Wang, Yunan Zhou, Zheng Liang, Naser Tavajohi, Baohua Li, Tao Li

**Affiliations:** ^1^ Engineering Laboratory for Next Generation Power and Energy Storage Batteries Graduate School at Shenzhen Tsinghua University Shenzhen Guangdong 518055 China; ^2^ Institute of Nuclear and New Energy Technology Tsinghua University Beijing 100084 China; ^3^ Department of Materials Science and Engineering Stanford University Stanford CA 94305 USA; ^4^ Department of Chemistry Umeå University Umeå 90187 Sweden; ^5^ Department of Chemistry and Biochemistry Northern Illinois University DeKalb IL 60115 USA

**Keywords:** flexible batteries, high Li^+^ conductivity, high LITN, safety, solid polymer electrolytes

## Abstract

Smart electronics and wearable devices require batteries with increased energy density, enhanced safety, and improved mechanical flexibility. However, current state‐of‐the‐art Li‐based rechargeable batteries (LBRBs) use highly reactive and flowable liquid electrolytes, severely limiting their ability to meet the above requirements. Therefore, solid polymer electrolytes (SPEs) are introduced to tackle the issues of liquid electrolytes. Nevertheless, due to their low Li^+^ conductivity and Li^+^ transference number (LITN) (around 10^−5^ S cm^−1^ and 0.5, respectively), SPE‐based room temperature LBRBs are still in their early stages of development. This paper reviews the principles of Li^+^ conduction inside SPEs and the corresponding strategies to improve the Li^+^ conductivity and LITN of SPEs. Some representative applications of SPEs in high‐energy density, safe, and flexible LBRBs are then introduced and prospected.

## Graphical abstract

Recent advancements in solid polymer electrolytes (SPEs) for Li‐based rechargeable batteries are summarized. The principles of Li‐ion conduction inside SPE and the corresponding strategies to improve Li‐ion conductivity and transference number are first reviewed. The representative applications of SPEs in high‐energy density, safe, and flexible batteries are then introduced and prospected.

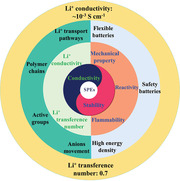

## 1 Introduction

**Figure 1:**
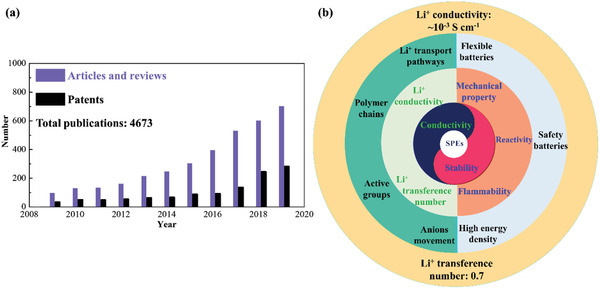
a) Publications and patents of SPEs‐based LBRBs from 2009 to 2019 (data source was obtained from Web of Science with solid polymer electrolyte and lithium battery as key words). b) Schematic presentation of the topics reviewed in this paper.

Li‐based rechargeable batteries (LBRBs) are widely used in applications from consumer electronics, vehicles, large‐scale energy storage, and integrated power systems to telecommunication equipment and applications^[^ ^1^, ^2^, ^3^, ^4^ ^]^ because of their high energy density and excellent cycling life.^[^ ^5^, ^6^ ^]^ However, safety concerns related to the use of flammable organic liquid electrolytes hinders LBRBs further development.^[^ ^7^, ^8^, ^9^, ^10^ ^]^ To tackle these safety concerns, solid‐state electrolytes (SSEs) are being employed to replace the liquid electrolyte, enabling LBRBs with excellent safety. Since Wright and coworkers reported the ionic‐conductive poly(ethylene oxide) (PEO) complexes with alkali metal salts in 1973^[^ ^11^ ^]^ and Armand summarized Li^+^ transport behaviors in the PEO‐based polymers,^[^ ^12^ ^]^ solid polymer electrolytes (SPEs) have been extensively studied (**Figure** **1**a).^[^ ^13^ ^]^ Their high chemical stability and wide electrochemical window^[^ ^14^ ^]^ make SPEs suitable for high‐performance LBRBs.^[^ ^15^ ^]^ However, the Li^+^ conductivity at room temperature and Li^+^ transference number (LITN) of PEO‐lithium bis(trifluoromethanesulfonyl)imide (LiTFSI) complex are only ≈10^−8^ S cm^−1^ and 0.2, respectively,^[^ ^16^ ^]^ and can only attain 10^−5^–10^−4^ S cm^−1^ and ≈0.5 at maximum, respectively, after further modification. This is the major reason why PEO‐based SPEs have not yet been widely commercialized.^[^ ^17^ ^]^

There were few papers that had reviewed the ion conduction mechanism and modification of PEO‐based SPEs prior to 2000,^[^ ^13^, ^18^, ^19^ ^]^ but research in this field notably accelerated after that (Figure 1a), with comprehensive summaries about SPEs of different polymers, comparison of different structure designs, and reports of progress, issues and future development in this field.^[^ ^20^, ^21^, ^22^, ^23^, ^24^ ^]^ This review mainly focuses on the principles and corresponding strategies concerning Li^+^ conductivity and LITN improvements of SPEs. The Li^+^ conduction principles of SPEs are discussed in terms of solvation sites, dissociation energy of Li^+^ from active groups, polymer chain movement, homogeneity of SPEs, Li^+^ transport pathways, and Li^+^ transport length. Improvements to LITN are described as well. We have also summarized available data about the Li^+^ conductivity and LITN as reported in the literature as well as predictions of future improvements. An overview of the use of SPEs in Li‐based high‐voltage, flexible batteries with improved safety is also presented. Finally, the future direction of research and development of SPEs has been summarized (Figure 1b).

## 2 Why SPEs?

The intensive research effort directed towards SPEs was mainly driven by their enhanced safety and applicability to batteries needing high energy density and good menchanical flexibility.^[^ ^25^ ^]^ Battery safety is the top priority, especially for high energy storage systems.^[^ ^26^ ^]^ The safety issues relating to state‐of‐the‐art LBRBs are associated with the use of flammable organic carbonate electrolytes. Liquid electrolytes offer high ionic conductivity and good interfacial contact with the electrode materials,^[^ ^27^ ^]^ however, their LITNs are low, being 0.2–0.3.^[^ ^28^ ^]^ Additionally, liquid electrolytes exhibit high leakage and reactivity.^[^ ^29^ ^]^ The exothermic reactions between the electrolyte and the electrode that occur during battery abuse conditions increase the overall battery temperature very quickly, triggering further electrolyte decomposition as well as generating flammable gases.^[^ ^30^ ^]^ This process is referred to as the notorious thermal runaway. The electrolyte becomes exposed to the air once the battery ruptures, and the resultant exothermic reaction causes smoke, fire and, in some cases, even explosions.^[^ ^31^ ^]^ In contrast, SPEs are (electro)chemically stable, which is beneficial in reducing or slowing down the heat generation of the batteries in thermal shock or thermal runaway situations.^[^ ^32^ ^]^ In relation to flexibility, consumer demands for convenient and personal smart electronics has led to the development of flexible and/or wearable devices, such as roll‐up displays, flexible phones, Apple Watch, Google Glass, electronic clothing, sensitive robotic skins, etc.^[^ ^33^ ^]^ Flexible devices even work when bent, folded, twisted, and stretched, and so their batteries have to as well.^[^ ^34^ ^]^ SPEs have a tremendous advantage over other technologies since the polymeric backbone of a SPE offers mechanical flexibility needed for such batteries.^[^ ^35^ ^]^

SPEs can replace not only the liquid electrolyte but also the traditional separator. This gives greater opportunities for exploring new electrode materials that increase battery energy.^[^ ^36^ ^]^ Most high‐energy active materials, such as layered lithium Ni–Mn–Co‐oxides (NMC) and lithium metal, cannot be used with traditional liquid electrolytes and show poor interface stability/compatibility.^[^ ^37^ ^]^ They also have a poor cycling profile and pose high severe safety risks. Thus, chemically and electrochemically stable SPEs appear to be promising for use in the next‐generation LBRBs using high‐energy electrodes.^[^ ^38^ ^]^

Considering the significant changes in battery technology and facilities, as well as the difficult issues around interface contact and charge space,^[^ ^10^, ^39^, ^40^ ^]^ the development of inorganic solid‐state electrolytes may still have a long way to go. However, the use of application of SPE‐based batteries in electric vehicles has been proved to be feasible over the last nine years.^[^ ^41^ ^]^ The ease of manufacture of SPEs makes them adaptable to state‐of‐the‐art battery technology and it is believed that such technology will dominate in the field of smart electronics in the future.

## 3 Overview of SPEs’ Historical Developments

**Figure 2:**
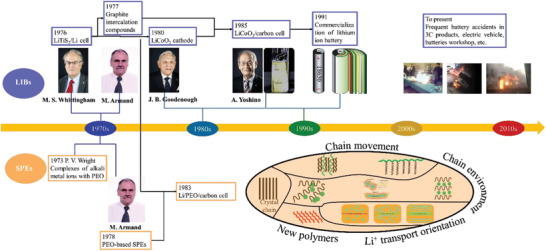
Development of LBRBs and SPEs. Photos of J. B. Goodenough, M. S. Whittingham, and A. Yoshino obtained from NobelPrize.org.^[^ ^248^ ^]^ Photo of M. Armand was obtained with permission.^[^ ^60^ ^]^ Copyright 2017, The Royal Society of Chemistry.

Development of SPEs has followed that of LBRBs (**Figure** **2**). In 1976, M. S. Whittingham developed the first LBRB, using Li*_x_*TiS_2_ as the cathode, but due to the safety issues of Li metal anode, it failed to be widely used.^[^ ^42^ ^]^ In 1980, Goodenough and coworkers synthesized a stable and high‐voltage LiCoO_2_ cathode capable of delivering several Li intercalation cycles, which significantly accelerated the development of LIBs.^[^ ^43^ ^]^ A remarkable cathode material was only half of the solution; an anode material that offered reliable and reversible Li intercalation was also in great demand. In 1977, Armand and Touzain demonstrated that graphite could be intercalated with ions.^[^ ^44^ ^]^ However, structural collapse occurred on the layered graphite due to solvent co‐intercalation, thus stopping the practical use of graphite. Then, in 1983, based on Wright, Armand, and other groups’ pioneering works,^[^ ^11^, ^12^, ^18^ ^]^ Yazami and Touzain tried to use SPEs to improve the compatibility of graphite anodes with electrolytes.^[^ ^45^ ^]^ The significance of this research was that it proved Armand's assumption that an appropriate electrolyte could create favorable conditions for successful and reversible graphite intercalation with Li^+^.^[^ ^18^ ^]^ Over the next two years, Yoshino et al. suggested using organic carbonate electrolytes for graphite||LiCoO_2_ cells.^[^ ^46^ ^]^ This design proved to be so successful that it was commercialized in 1991 by Sony Corporation.^[^ ^47^ ^]^

From 2004, safety issues, characterized by spontaneous ignition or explosion, attracted increasing attention. SPEs are ideal alternatives to flammable organic liquid electrolytes as they reduce the combustibility of the battery because they are non‐volatile and almost nonflammable. However, PEO‐based SPE has very low room temperature ionic conductivity, which needs to be above ≥10^−4^ S cm^−1^ to meet practical requirements.^[^ ^48^ ^]^ After thorough and time‐consuming research, the mystery of Li^+^ conductivity in SPEs was gradually solved. The polymer chain movement, dissociation energy of Li^+^ from polymer chains, and Li^+^ transport path are the key factors,^[^ ^49^ ^]^ driving four strategies to improve the ionic conductivity of PEO‐based SPEs: 1) reduce the crystallinity of SPEs and improve the polymer chain movement;^[^ ^50^ ^]^ 2) employ inorganic fillers (with intrinsically good ionic conductivity) and other additives (such as plasticizer) to make the SPE structure more uniform as well as to increase free Li^+^ in SPEs;^[^ ^51^, ^52^ ^]^ 3) orientate the Li^+^ transport path to minimize the transport length of Li^+^ in SPEs, and 4) develop SPEs with optimized frameworks of ion‐conducting groups, which can be derived from PEO, polyetherimide (PEI), polyacrylonitrile (PAN), polycarbonate, polyborane, and so on.^[^ ^53^, ^54^, ^55^, ^56^ ^]^ This latter strategy will be further discussed in details in the part of Li^+^ conductivity improvement. A high LITN is essential for a high‐performance battery. A low LITN creates concentration overpotential^[^ ^57^ ^]^ and reduces the energy and power efficiency of the entire battery.^[^ ^58^ ^]^ Liquid electrolytes and pure PEO‐based SPEs exhibit relatively low LITNs. Specifically, McCloskey and coworkers found that a higher LITN is beneficial to attaining a higher state of charge (SOC) of batteries.^[^ ^57^ ^]^ This issue will be discussed in later sections of this review. To obtain an electrolyte with an ideal LITN, it is necessary to limit the anion transport during battery cycling, so for SPEs that means immobilizing the anions in the polymer.^[^ ^59^ ^]^ In fact, a single ion SPE was reported in 1984, and later developed further by Armand and coworkers.^[^ ^60^, ^61^, ^62^ ^]^ It was an essential part of SPE‐dedicated research and development. Low ionic conductivity of SPEs at room temperature means that SPE‐based batteries are usually operated at elevated temperatures in practical applications. SPEs are being widely researched with a view to increase their ionic conductivity, making batteries safer, fabricating flexible batteries, etc.^[^ ^20^, ^24^, ^63^, ^64^ ^]^ Although many SPE‐based batteries appeared on the market around the year 2000, they were not used as extensively as first hoped. After 2010, due to serious battery safety issues, the interest in SPE‐based batteries exponentially increased. The most successful SPE battery application was for an electric vehicle containing LiFePO_4_/PEO/Li battery cells.^[^ ^41^ ^]^ However, this battery could only operate at 60–80 °C. Some researchers developed cells based on SPEs with small molecules for drone‐related applications.

## 4 Criteria for SPEs

**Figure 3:**
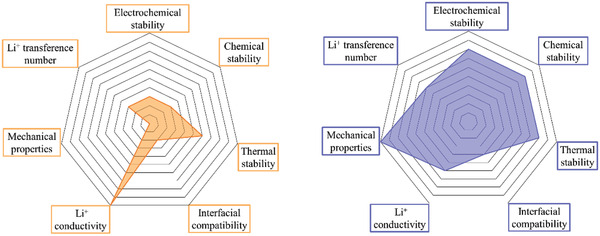
Radar plots comparing electrochemical/chemical/thermal stability, interfacial compatibility, mechanical properties, Li^+^ conductivity, and Li^+^ transference numbers of liquid electrolytes (left) and SPEs (right).

Although improving Li^+^ conductivity is currently the most important goal in the research on SPE, other properties, such as stability, interface compatibility, mechanical strength, LITN, etc., also need to be considered when designing SPEs for practical applications. The following sections describe method of improving these properties and the corresponding comparison to liquid electrolyte. It is showed that SPEs are superior to liquid electrolytes in terms of electrochemical, chemical, and thermal stability.^[^ ^20^ ^]^ They also offer more favorable LITN, mechanical strength, and chemical interfacial compatibility than traditional liquid electrolytes, which makes them suitable for high‐energy‐density, safe, and flexible batteries (**Figure** **3**).^[^ ^65^, ^66^ ^]^ Therefore, if Li^+^ conductivity could be increased to the desired levels, SPEs would be a significant development for the entire battery industry, improving on organic liquid electrolytes.

### 4.1 Li^+^ Conductivity

SPEs with low Li^+^ conductivity can only allow the battery to operate at a low charge/discharge rate. Thus, it could take several days for such a battery to charge fully. Additionally, the allowable discharge rate would be insufficient for a device to operate. Therefore, to ensure normal use of SPE‐based batteries, Li^+^ conductivity needs to exceed 10^−4^ S cm^−1^.^[^ ^48^ ^]^

### 4.2 Electrochemical Stability

The electrochemical stability determines whether SPEs can be practically used for batteries. The charge and discharge characteristics of electrode materials should be within a specific voltage range. The voltage range across which SPEs are stable should cover the voltage range of the selected electrode materials, otherwise, SPEs undergo side reactions and are not capable of maintaining normal battery operation. Current commercial anodes are composed of graphitic/carbonaceous materials with the Li^+^ insertion potential below 0.1 V versus Li/Li^+^.^[^ ^67^ ^]^ The maximum voltage of state‐of‐the‐art commercial cathode materials (e.g., LiFePO_4_, LiCoO_2_, LiNi_1/3_Co_1/3_Mn_1/3_O_2_) is less than 4.3 V.^[^ ^68^ ^]^ However, the ideal battery chemistry consists of a Li metal anode and the charge window of future cathode materials will likely increase to greater than 4.5 V.^[^ ^68^ ^]^ Therefore, it would be ideal for a SPE to be stable between 0 and 5 V versus Li/Li^+^.

### 4.3 Chemical Stability

The chemical stability of SPEs profoundly influences their ease of fabrication and electrochemical performance. Materials with poor chemical stability require challenging manufacturing processes and cumbersome battery technology. It has been proved that a chemically unstable SPE will, very likely, be poorly compatible with active electrode materials, generating gas and harmful small molecules which are detrimental to the battery safety and service life.^[^ ^69^ ^]^ The cathode materials are most active in the charged state and, if SPEs are chemically unstable, can catalyze SPE decomposition.^[^ ^14^, ^70^ ^]^

### 4.4 Thermal Stability

The battery separator plays an important role in isolating the cathode and anode and preventing the battery from short‐circuiting. If SPEs successfully replace traditional electrolytes and separators, they are still required to have excellent thermal stability so that they do not shrink or suffer severe shape/volume change below 150 °C.^[^ ^71^ ^]^ Ideally, at 200 °C, the allowable shrinkage is <10%.^[^ ^72^ ^]^ No melting should be exhibited to ensure the structural integrity of the battery, even at elevated temperatures.

### 4.5 Li^+^ Transference Number

SPEs formed by dissolving Li salt in polymer hosts are generally dual‐ion conductors. In this case, both Li^+^ and its counter anions are mobile. Li^+^ is typically less mobile than its anionic counterpart since its motion is coupled with the Lewis basic sites of the polymer matrix. The ratio of migrating Li^+^ to all migrating ions including anions in the electrolyte is defined as the LITN. During charge/discharge cycling, only migrating Li^+^ contribute to the performance of the battery. At low LITN, local polarization is serious and makes Li^+^ deposition uneven. As a result, the cycle life and power density of the battery are degraded.^[^ ^73^ ^]^ Therefore, the higher LITN of an SPE, the better the battery performance is. Overall, the ideal LITN is equal to 1.^[^ ^57^, ^60^ ^]^

### 4.6 Mechanical Strength

Mechanical strength is essential when considering practical SPE applications.^[^ ^74^ ^]^ SPEs that are too brittle or too hard might exhibit poor compatibility with the electrodes. Liquid electrolytes possess excellent flowability and can fully infiltrate the electrode materials. SPEs with poor direct contact with the electrode surface will not provide adequate charge transfer.^[^ ^75^ ^]^ Over the long term, this could increase structural defects in a battery, which is the main reason for the degraded battery life.^[^ ^76^ ^]^ SPEs that are too soft and sticky is also a disadvantage because special processing would be needed, which, in turn, would increase the battery fabrication cost.

## 5 Strategies to Improve Li^+^ Conductivity

In this section, the mechanism of Li^+^ transport in polymer chains and SPEs is described. Then, the key factors influencing Li^+^ transport are outlined. From this, we briefly review improvement of Li^+^ conductivity from the perspective of increasing solvated Li^+^, improvement of dissociation of the Li‐active group bond and polymer chain movements, creation of uniform SPEs, introduction of new Li^+^ transport pathways, and the control of Li^+^ transport length. At the end of this section, a brief summary of improvement strategies for Li^+^ conductivity reported in current literature is presented.

### 5.1 Li^+^ Transport Mechanisms in Polymer Chain and SPEs

**Figure 4:**
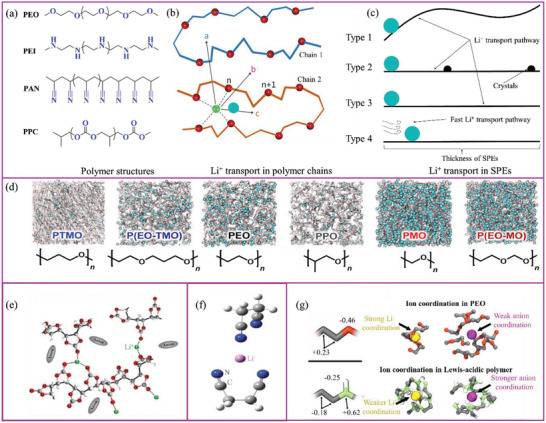
a) Active groups (blue color) and their representative polymers. b) Li^+^ transport mechanisms in polymer chains a, b, and c represent Li^+^ transport mechanisms by the interchain diffusion, shift, and intrachain diffusion, respectively). Li atom: green; O atoms: red. Reproduced with permission.^[^ ^88^ ^]^ Copyright 2015, American Chemical Society. c) Schematics of Li^+^ transport mechanisms in SPEs. Type 1: Li^+^ transport in uniform SPE region where a long internal transport distance/route is required; Type 2: Li^+^ transport in non‐uniform SPE, where both amorphous and crystalline region are present, impeding its fast conduction; Type 3: ideal Li^+^ transport; Type 4: rapid Li^+^ transport under external promotions. d) The density of Li^+^ solvation sites in different polymers obtained by molecular dynamics. Green circles represent the density of solvation sites. Reproduced with permission.^[^ ^85^ ^]^ Copyright 2016, American Chemical Society. e) Possible interaction of Li^+^ with carbonate groups. Reproduced with permission.^[^ ^90^ ^]^ Copyright 2017, Wiley‐VCH. f) Spatial structure of Li^+^ interacting with two succinonitrile molecules according to simulations. Reproduced with permission.^[^ ^93^ ^]^ Copyright 2018, American Chemical Society. g) The charges of PEO and Lewis‐acidic‐based polymer obtained by simulations and the coordination structures of Li^+^ and anions in PEO and Lewis‐acidic‐based polymer. C atom: grey; O atoms: red; B atom: green; Li atoms: yellow. Reproduced with permission.^[^ ^87^ ^]^ Copyright 2019, American Chemical Society.

To improve the Li^+^ conductivity in SPEs, it is important to understand its conduction mechanism. At the polymer chain level, polymers containing polar functional groups (such as —O—, —B—, —N—, C=O, C≡N, C—S—, C—(O=S=O)—) can dissolve Li salts and form polymer–salt complexes.^[^ ^13^, ^77^, ^78^, ^79^ ^]^ Among the polymers containing the polar groups mentioned, PEO, PEI, PAN, and polypropylene carbonate (PPC) all showed satisfactory Li salt solvation (**Figure** **4**a) capability. Li^+^ is coordinated by the ether oxygen atoms of the PEO chain and moves through the coupling/decoupling of Li–oxygen bonds.^[^ ^24^, ^80^ ^]^ At the polymeric chain level, Li^+^ transport occurs either as an interchain diffusion, shift, or intrachain diffusion (routes a, b, and c, respectively, in Figure 4b), while the intrachain diffusion is the major contributor to the Li^+^ conductivity in the case of PEO‐based SPEs.^[^ ^80^ ^]^ Through the mechanism of Li^+^ transport on the polymer chain, it can be seen that the amount of polymer solvated Li^+^, the dissociation energy of Li—O bonds, and the mobility of the polymer chain are the main factors affecting conduction.

SPEs with thicknesses up to tens of micrometers are made up of a large quantality of polymeric molecular chains and Li salts, which are the basis for Li^+^ transport in SPEs. Li^+^ transport pathways in SPEs are generally longer than the thickness of the SPE film, which is up to tens of micrometers between the electrodes and hundreds of micrometers in the porous electrode. Controlling Li^+^ transport across SPEs utilizing a short path is important (Types 1 and 3 in Figure 4c). For example, Park and coworkers found that the enhanced segregation strength of lamellar phase separation of copolymer‐based SPEs with a Li:EO of 0.02 showed better Li^+^ conductivity.^[^ ^81^ ^]^ The homogeneity of SPEs, achieved, for example, by destroying the crystallinity of polymer, ensures smooth Li^+^ transport (Type 2 and 3 in Figure 4c).^[^ ^82^ ^]^ This is due to the intrinsically low ionic conductivity of polymers, although SPEs are mostly amorphous but still exhibit low Li^+^ conductivity (≤10^−4^ S cm^−1^ at ambient temperature). Thus, new fast Li^+^ transport pathways inside SPEs are required and are a defining aspect of the practical applicability of the resulting SPEs (Types 3 and 4 in Figure 4c).^[^ ^83^ ^]^ Yet, the naturally‐formed Li^+^ transport pathway are associated with the preparation and the filler dispersion in the SPEs, which results in different tortuosity. Hence, the orientation of Li^+^ transport pathways, which determines the transport length of Li^+^ in SPEs, has been studied closely.^[^ ^84^ ^]^

### 5.2 Li Salt Solvation in Polymer Chains

The amount of solvated Li^+^ is influenced by the density of active groups and the polymer molecular structure. Polymers with less dense active groups generally show fewer Li solvation sites. For example, poly(trimethylene oxide) (PTMO) exhibits fragmentary solvation sites whereas poly(methylene oxide) (PMO) exhibits the greatest amount (Figure 4d).^[^ ^85^ ^]^ In addition, polymer structure has a significant influence on Li solvation sites in SPEs. Poly(propylene oxide) (PPO) and PTMO exhibit the same element ratio of C:O in their polymer chains yet PPO has more sites than PTMO. The use of copolymers is an effective strategy for improving Li solvation sites. PEO‐based copolymer with PTMO as the repeating unit (poly(ethylene oxide‐alt‐trimethylene oxide) (P(EO‐TMO))) has a similar site density to that of PEO while PEO copolymerized with PMO to form poly(ethylene oxide‐alt‐methylene oxide) (P(EO‐MO)) has numerous Li solvation sites. It is worth noting that a lower density of Li solvation sites may limit Li^+^ diffusion in the polymer. Efficient and facile Li^+^ diffusion can be achieved through frequent ion hopping over a shorter distance. The ideal Li^+^ transport method would be to break one or two lithium–oxygen solvation species at every hop, meaning less energy consumption compared to hopping over longer distances.^[^ ^86^ ^]^

### 5.3 Dissociation of Li Ions with Active Groups in Polymer Chains

The dissociation energy of Li ions with active groups is the limiting step for Li^+^ transport in polymer chains,^[^ ^87^ ^]^ and may be related to the Li^+^ coordination number as well as the interaction between Li^+^ and active groups. In general, for the case of PEO, Li may be coordinated by 4.5 oxygen atoms with a distance around 2.55 Å (Figure 4e).^[^ ^88^ ^]^ The helical distortion of its chemical structure indicates and confirms the presence of strong coordination of Li^+^ in PEO.^[^ ^87^ ^]^ Molecular dynamics simulations have shown that each Li^+^ coordinates 5.5 oxygen atoms, which agrees with the experimentally‐obtained data very well. The entire complex consists of 4 carbonyl oxygen atoms and 1–2 oxygen atoms from the anion of Li salt in carbonate polymers.^[^ ^89^ ^]^ Due to the steric hindrance effect, Li^+^ favors interactions with C=O instead of —O—C=O groups.^[^ ^90^ ^]^ It was also proposed that the carbonyl (C=O) groups might still weakly interact with Li^+^ compared with the interaction of Li^+^ and oxygen.^[^ ^91^ ^]^ Tominaga et al. proposed the possible interaction between Li^+^ and a single C=O group. In addition, based on their assumptions, this interaction was believed to lower the Li^+^ dissociation barrier and thus enable Li^+^ to migrate faster through the segmental motion of poly(ethylene carbonate) (PEC) chains.^[^ ^92^ ^]^ The interaction of cyano groups and Li^+^ may be the main route for Li^+^ transport in PAN‐based SPEs. The Li^+^ coordination number of the Li^+^–CN group was found to be 2–3 (Figure 4f).^[^ ^93^ ^]^ Recently, it has been shown that the PAN‐LiClO_4_ system containing cyano‐based polymeric chains is indeed, a very promising alternative with a conductivity of 10^−4^ S cm^−1^ at ambient temperature for PEO‐based SPEs.^[^ ^94^ ^]^ Cui and coworkers further modified cyano‐based SPEs by designing composite polymers containing different functional groups and/or additives, improving the Li^+^ conductivity to ≈10^−3^ S cm^−1^ at ambient temperature.^[^ ^21^ ^]^ The Li^+^ transport mechanism in alkyl imines is similar to PEO and the same ionic species are formed in the Li salt–PEI system which is analogous to that in the PEO system. Frech and coworkers compared Li^+^ conductivity in PEI, poly(propylenimine) (PPI), poly(*N*‐methylethylenimine) (PMEI), and poly(*N*‐methylpropylenimine) (PMPI) using lithium triflate (LiTf).^[^ ^53^, ^95^, ^96^, ^97^ ^]^ The Li^+^ conductivity of PPI‐based SPEs at room temperature is relatively low (≈10^−7^ S cm^−1^). However, it increases by three orders of magnitude at 70 °C. Li^+^ conductivity in PEI‐based SPEs is somewhat irregular as Li salt dissociation in PEI‐based SPEs depends on the salt concentration, but, at the same time, their amorphous phase content is determined not only by the Li salt content but also by the temperature. PMPI‐based SPEs exhibit a linear relationship between Li^+^ conductivity and the temperature. The temperature is a more significant factor in defining Li^+^ mobility than Li salt concentration. At a PMPI:LiTf ratio equal to 10, Li^+^ conductivity was the highest.^[^ ^53^ ^]^ Despite alkyl imines being favorable compounds, their currently low Li^+^ conductivity still makes them inadequate as SPEs. Recently, Miller and coworkers demonstrated (using molecular dynamics simulations) that polymers with Lewis‐acid‐base active groups are excellent alternatives to PEO‐based SPEs as they offer high Li^+^ conductivity.^[^ ^55^, ^87^ ^]^ In conventional PEO or other similar SPEs, Li^+^ strongly interacts with active groups to form cation‐polymer interactions, which leads to relatively sluggish Li‐ion diffusion and rapid anion diffusion, therefore resulting in low LITN. Replacing PEO with a Lewis acidic polymer can reverse this relationship, eventually leading to an increase in Li^+^ diffusion while preserving the same salt solubility. (Figure 4g). In 2001, Johansson showed that a —C—S— group in a polymer has a lower binding energy (433 kJ mol^−1^) to Li^+^ than that of —C—N— and —C—O— groups.^[^ ^98^ ^]^ There is no steric hindrance for the coordination of Li^+^ in a —C—S— group‐based polymer resulting in the facilitated segment motion of the polymer. However, the solubility of lithium salts in polythioethers is low, therefore, the —C—S— group generally becomes incorporated into a —C—O— group based polymer for SPEs. Tew and coworkers synthesized a series of —C—S— group based polymer with low glass transition of −50 to −75 °C, which showed high room temperature ionic conductivity of 10^−5^–10^−4^ S cm^−1^.^[^ ^99^ ^]^ Guo and coworkers demonstrated that a —C—S— group based‐polymer enabled LiFePO_4_/SPE/Li battery delivered a specific capacity of 140 mAg^−1^ at 0.05 C with a capacity retention of 99% after 100 cycles.^[^ ^100^ ^]^

### 5.4 Movement and Homogeneity of Polymeric Chains in SPEs

Understanding how polymer chain movement increases SPE ionic conductivity is also very important. Li^+^ transport is directly linked to the motion of the polymer chain segments and mainly occurs in the amorphous part of an SPE. Unmodified polymers, such as PEO or PAN, are semicrystalline and possess very low ionic conductivity at room temperature.^[^ ^21^, ^50^ ^]^ At higher temperatures, Li^+^ conductivity of PEO‐based SPEs can be higher (up to ≈10^−4^ S cm^−1^). Therefore, most research in the past focused on developing fully amorphous SPEs. Recent research has revealed that Li salt incorporation decreases PEO crystallinity. At an EO/Li^+^ ratio equal to eight, the resulting SPEs are mostly amorphous but still show low Li^+^ conductivity.^[^ ^101^ ^]^ Thus, apart from achieving the appropriate amorphous content for SPEs, Li^+^ transport inside the polymer chains is more critical for determining the practicality of the resulting SPEs. For example, poly(trimethylene carbonate) (PTMC) and PPC‐based SPEs are 100% amorphous up to 100 °C. Cui and coworkers suggested that the high polymer segmental mobility is responsible for increased Li^+^ conductivity in PPC‐based SPEs, being ≈3.0 × 10^−4^ S cm^−1^ at 20 °C.^[^ ^65^ ^]^ This value is significantly higher than that for PEO.

**Figure 5:**
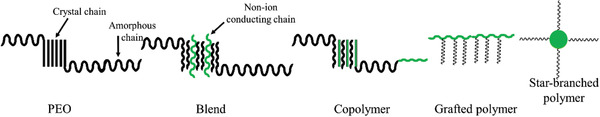
Strategies to decrease the crystallinity of a polymer: blending, copolymerization, grafting, and branching.

Reducing the polymer's crystallinity is an effective way to enhance segmental mobility thus increasing ion conduction. A variety of polymer blends, along with blocked, grafted, and hyperbranched copolymers based on PEO, were designed to decrease the crystallinity of the resulting backbone, and thus increase polymer chain movement (**Figure** **5**).^[^ ^102^, ^103^, ^104^, ^105^, ^106^ ^]^ The ionic conductivity of branched PEO‐based SPEs is ≈10^−5^ S cm^−1^ (which is significantly higher than the ionic conductivity of linear PEO‐based SPEs) because they have a lower degree of crystallinity, possess PEO chains that move easily, and can accommodate higher concentrations of dissolved Li salts.^[^ ^107^ ^]^ Blending is also a very popular method to improve SPE performance because of the synergetic effect and interactions between the two or more materials. For example, hydrogen bond formation can decrease SPE crystallinity and positively affect its Li^+^ conductivity.^[^ ^108^ ^]^ Copolymers and grafted polymers are self‐assembling compounds forming ion‐conducting chains acting as Li^+^ transport channels.^[^ ^109^ ^]^ A list of possible branched SPEs is quite diverse because of the numerous possible combinations of backbones and branching chains, as well as polymer cores. Phosphates, boron‐containing compounds, polyhedral oligomeric silsesquioxane, and even organic nanoparticles, have all been utilized as cores.^[^ ^110^, ^111^, ^112^, ^113^ ^]^

Designing lower molecular weight chains is another way to enhance segmental mobility. A variety of alternative polymeric materials were reported in the literature, which mostly focused on modification of low molecular weight polymer chains that exhibited fast mobility and were capable of forming complexes with Li salts. Polyphosphazene, polysiloxane, or polyacrylic acid, acting as the polymer backbones, can be easily grafted onto other more active chains.^[^ ^114^, ^115^ ^]^ Boroxine was successfully used as a core linking polymer segment.^[^ ^116^, ^117^, ^118^ ^]^ Polystyrene‐b‐PEO (PS‐PEO) is the most widely researched copolymer capable of self‐assembling (at certain PEO:PS ratios) into a nanostructure.^[^ ^119^ ^]^ During SPE design, polymer chains with low molecular weights can be used as side chains.^[^ ^103^, ^120^, ^121^, ^122^ ^]^ Ethylene glycol (EG) fragments of poly(ethylene glycol) (P(EG)*_n_*) with *n* > 9 tend to crystallize, which decreases the ionic conductivity of the whole system.^[^ ^122^ ^]^ In its free form, the Li^+^ conductivity of P(EG)_9_ at room temperature is high (≈10^−3^ S cm^−1^) because of the unrestricted motion of the EG segments. However, P(EG)_9_ and other low molecular weight PEGs, are liquid in their original form and are, thus, not suitable as battery SPEs. Fortunately, these PEGs can be grafted onto other polymer backbones that have suitable mechanical properties. In this case, Li^+^ conductivity of P(EG)_9_‐based SPEs can be reduced relative to the unbound P(EG)_9_ but is still at ≈10^−5^ S cm^−1^ order of magnitude. When P(EG)_5_ was used as a side chain, the Li^+^ conductivity of the resulting SPE dropped significantly because these short chains limited Li^+^ hopping.

Some cross‐linked polymers can also be highly amorphous.^[^ ^123^, ^124^, ^125^ ^]^ He and coworkers (2012) demonstrated excellent miscibility of methoxy polyethylene glycol (350) monoacrylate and polyethyleneglycol (200) dimethacrylate, which was beneficial for a fast cross‐linking reaction to obtain a polymer with a perfect interpenetrating polymer network (IPN).^[^ ^123^ ^]^ The typical room temperature ionic conductivity of IPN‐LiClO_4_ SPEs is ≈1.5 × 10^−5^ S cm^−1^. These materials also demonstrate excellent thermal stability up to 270 °C and acceptable charge/discharge performance (as was demonstrated for Li/SPEs/LiCoO_2_ coin cells). Plasticization is one of the most common ways of increasing the Li^+^ conductivity of an SPE. The purpose of adding plasticizer is to increase the amorphous phase content in the SPEs and increase its chain motion. The plasticizer also promotes the dissociation of ion pairs and, as a result, increase the number of free Li^+^ available for charge transport. Typical plasticizers are organic solvents, ionic liquids, etc.^[^ ^22^, ^126^, ^127^, ^128^, ^129^, ^130^ ^]^ Succinonitrile (SN) is the most‐commonly used plasticizer to improve SPE properties.^[^ ^21^, ^131^ ^]^ Its plastic crystal transition is at −40 °C, and it can dissolve Li salts at room temperature. Fan et al. revealed that SPEs containing ≈50% of SN were completely amorphous.^[^ ^132^ ^]^ When SN was added into PEO‐based SPEs, both SN and PEO interacted with Li salt. Thus, in this case, the plasticizer not only changed the polymer's micro‐environment but also promoted Li salt dissociation. Even the addition of small SN doses increased the Li^+^ conductivity of SPEs because the SPE crystallinity had decreased. Tominaga and coworkers showed that when ionic liquid was added into SPE, the segmental motion of PEC was improved.^[^ ^133^ ^]^ He and coworkers synthesized a macromolecule plasticizer for IPNs‐based SPEs using PEG350 and PEG200 as the precursors. When the resulting macromolecular compound was added to SPEs, the samples showed higher Li^+^ conductivity: SPE containing 5 wt% of IPN‐LiClO_4_ demonstrated ionic conductivity up to 6.06 × 10^−5^ S cm^−1^.^[^ ^134^, ^135^, ^136^ ^]^ However, plasticizer addition to SPEs results in the loss of mechanical properties and the formation of undesired gel polymers.

### 5.5 Li^+^ Transport Pathways in Bulk SPEs

When material design alone cannot fully achieve the desired Li^+^ conductivity, novel strategies can be explored at the bulk conduction level, such as the introduction of inorganic additives into the SPEs. Inorganic additives for SPEs are typically divided into active (or capable of conducting Li^+^ themselves) or inactive (those that provide a favorable environment for Li^+^ transport in a polymer matrix). Inactive additives can absorb anions on their surfaces and form layers capable of interacting with the polymer chain and Li^+^. The effects of inactive additive incorporation can be two‐fold: enhancement of free Li^+^ mobility and suppression of polymer recrystallization, which also makes SPEs homogeneous.

#### 5.5.1 Inactive Additives

Since the first report of SPEs modified with *α*‐Al_2_O_3_ nanoadditive by Weston and Steele, research on how different inorganic additives affect SPE performance has intensified.^[^ ^137^, ^138^, ^139^ ^]^ Inactive additives often suppress the polymer's recrystallization, as shown by Scrosati et al., who dispersed inorganic nanoparticle additives in PEO‐based SPE.^[^ ^140^ ^]^ They observed changes in the recrystallization kinetics and an increase in Li^+^ conductivity while the amorphous state of the SPE remained the same. Typically, an amorphous state of unmodified PEO changes with temperature. However, with additives, the semi‐crystalline PEO matrix becomes amorphous, and this state does not change with temperature. This effect can be explained by a large surface area and the Lewis‐acid characteristics of the additives that prevent PEO chain reorganization. The Li^+^ conductivity of an *α*‐Al_2_O_3_‐filled PEO‐LiClO_4_‐SPE was 10^−5^–10^−3^ S cm^−1^.^[^ ^141^ ^]^

**Figure 6:**
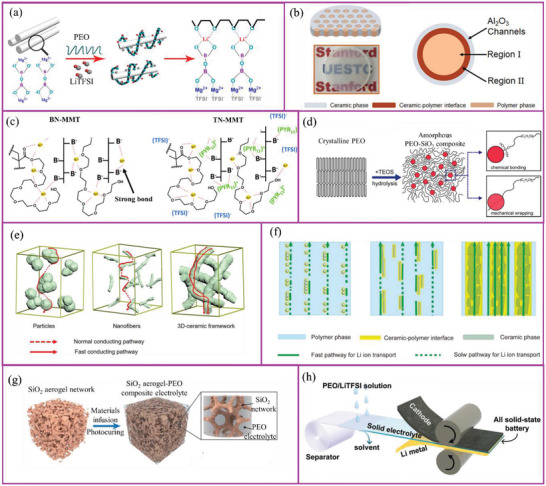
a) Incorporation of inactive additives to enhance ion transport. Reproduced with permission.^[^ ^142^ ^]^ Copyright 2018, American Chemical Society. b) Schematics of Li^+^ conducting channels in composite SPE. Reproduced with permission.^[^ ^144^ ^]^ Copyright 2018, American Chemical Society. c) Schematic presentation of the inactive additives (montmorillonite) interaction with the polymer matrix and effect of its dissociation energy on the SPE ion transport. The boron atom shows strong binding energy with Li^+^. When (PYR11)^+^, a kind of ionic liquid added into the SPE, it tends to interact with anions and release more free Li^+^. Reproduced with permission.^[^ ^146^ ^]^ Copyright 2017, The Royal Society of Chemistry. d) Distribution of inactive additives (SiO_2_) in PEO‐based SPE. Reproduced with permission.^[^ ^152^ ^]^ Copyright 2015, American Chemical Society. e) Schematics of conducting pathways for nanoparticle, nanowire (or nanofiber) and continuous 3D structures filled in SPE. Reproduced with permission.^[^ ^249^ ^]^ Copyright 2019, American Chemical Society. f) Schematics of conducting pathways for vertically aligned nanoparticle, nanowire (or nanofiber) and continuous fiber structures filled in SPE. g) Schematic showing the synthetic procedures of the SiO_2_‐aerogel‐reinforced SPE. Reproduced with permission.^[^ ^250^ ^]^ Copyright 2018, Wiley‐VCH. h) Schematic illustration of the fabrication of porous film based SPEs. Reproduced with permission.^[^ ^156^ ^]^ Copyright 2019, Wiley‐VCH.

According to the Lewis acid‐base theory, incorporation of inactive additives into SPEs can increase the free Li^+^ content; acidic additives are more effective at increasing conductivity. Some research groups suggested that a stronger affinity between anions and acidic groups on the nano‐oxide surfaces helps to dissociate Li salts and increase content and transport of free Li^+^ by forming conductive pathways at the interface of nanoparticle/polymer chain.^[^ ^142^ ^]^ Some oxide nanoparticles can absorb anions and form space charge layers, which might interconnect with the charge pathways created by the polymer chains.^[^ ^143^ ^]^ Such an arrangement might result in the formation of 3D percolative spaces around the polymer‐oxide composite, which would ultimately increase Li^+^ mobility (**Figure** **6**a). Cui and coworkers showed that there are two Li^+^ conducting channels in composite SPE, namely, region I polymer matrix and region II ceramic−polymer interface.^[^ ^144^ ^]^ The latter shows a higher Li^+^ conductivity of >10^−3^ S cm^−1^ (Figure 6b).

Immobilization of anions in SPEs suppresses the electrode polarization and reduces ionic diffusion resistance at the electrode surface. In PAN, Li^+^ ions migrate through the polymer/inactive additive interface forming complexes by weakly coordinating with the 1) C≡N PAN groups, 2) Lewis‐base sites on the additive surfaces, or 3) vacancies.^[^ ^145^ ^]^ All of these provide shorter paths for the Li^+^ to migrate along. However, the attraction and the forces produced between the inactive additives and Li^+^ need to be considered as well. Too strong an interaction might completely block Li^+^ transport. For example, negatively charged montmorillonite (MMT) surface negatively affected Li^+^ transport when incorporated into a PEO‐based SPE. However, when an additional ionic liquid (capable of interacting with anions on the MMT surface) was added to the system, MMT surface interaction with the Li^+^ in the system became weaker, which enhanced Li^+^ transport (Figure 6c).^[^ ^146^ ^]^

Not only traditional inorganic but also some novel organic inactive additives were added in an attempt to improve Li^+^ transport in SPEs. Ardebili and coworkers added clay particles functionalized with carbon nanotubes (CNTs) into SPEs.^[^ ^147^ ^]^ CNTs attached to the clay surface suppressed PEO chain reorganization allowing the Li^+^ conductivity of the clay‐CNT‐PEO‐based SPE to achieve a conductivity equal to ≈2 × 10^−5^ S cm^−1^ with only 10% of clay‐CNTs by weight added to the system. Gerbaldi et al. developed a PEO‐based nanocomposite SPE by adding aluminum benzenetricarboxylate.^[^ ^148^ ^]^ The resulting material showed Li^+^ conductivity two orders of magnitude higher than unmodified PEO and a LITN equal to 0.55. Another study reported the addition of Y_2_O_3_‐doped ZrO_2_ to introduce Lewis acid vacancies to bind with anions of Li salt and to liberate Li^+^. The addition of 7 mol% of Y_2_O_3_‐doped ZrO_2_ nanowires resulted in the highest ionic conductivity reported for this inorganic SPE additive, which was equal to 1.07 × 10^−5^ S cm^−1^ at 30 °C.^[^ ^149^ ^]^

Undoubtedly, to influence Li^+^ transport, inactive additives need to be well‐dispersed in the SPEs to shorten the Li^+^ transport pathways. The existing methods of filled SPE preparation are mostly based on mixing the inorganic particles with the host polymer in a solution. Agglomeration of nanoadditives results in their uniform distribution in the SPE matrix.^[^ ^150^ ^]^ He and coworkers paid close attention to the agglomeration problem during the incorporation of nano‐TiO_2_ into the SPE matrix.^[^ ^151^ ^]^ Cui and coworkers reported the existence of a considerable number of crystallized polymer regions in PEO even after nanoparticles were homogeneously incorporated into the SPE matrix (Figure 6d).^[^ ^152^ ^]^ They developed a PEO‐based composite containing monodispersed ultrafine SiO_2_ (with average particle sizes of ≈12 nm) synthesized using an in situ hydrolysis of tetraethyl orthosilicate in a PEO solution. They showed that the nano‐SiO_2_ directly reacted with PEO chains, which helped their homogeneous distribution and, at the same time, suppression of PEO recrystallization. Compared to the ex‐situ added nano‐SiO_2_, the in situ SiO_2_ addition improved the Li^+^ conductivity of the resulting SPEs by one order of magnitude: at 30 and 60 °C the conductivity of the resulting SPE‐composite was equal to 4.4 × 10^−5^ and 1.2 × 10^−3^ S cm^−1^, respectively.

Although inactive additives can destroy the crystallinity inside SPEs and improve the Li^+^ transport performance in the polymer phase, ordinary nanoparticles tend to agglomerate or disperse non‐uniformly in SPE. Compared with nanoparticles, nanofibers have a longer one‐dimensional size and can form a longer Li^+^ transport path. Nevertheless, nanofibers tend to be arranged horizontally in SPEs. Therefore, forming a continuous additive phase in the SPE helps improve the uniformity of Li^+^ transport (Figure 6e). Ideally, an additive‐polymer vertical interface is formed in the SPE (i.e., the additives are arranged vertically in the SPEs), which results in more efficient Li^+^ transport in SPEs (Figure 6f). There are many types of additive structures that can be used to create vertical Li^+^ paths in SPEs, including nanoparticles, nanofibers, 2D materials, and even inorganic materials with vertically aligned pores. It is worth noting that the design of these composite SPEs is suitable for both inactive additive‐filled and active additive‐filled SPEs.

To distribute inactive additives in SPEs continuously, the simplest way may be to prepare the inactive additives into a three‐dimensional continuous skeleton in advance, and immerse the polymer in the skeleton to form a continuous ion transport channel while enhancing the mechanical strength of the SPE.^[^ ^153^ ^]^ Cui and coworkers using a stiff mesoporous SiO_2_ aerogel as the backbone and PEO/LiTFSI as the Li^+^ conducting phase fabricated a high‐performance SPE, with a high Li^+^ conductivity of 6 × 10^−4^ S cm^−1^ at 30 °C (Figure 6g). The LiFePO_4_/SPE/Li cells delivered a stable capacity of 105 mAh g^−1^ within 200 cycles.^[^ ^154^ ^]^

1D nanofiber additives are equivalent to nanoparticles extending along one dimension, so they greatly improve the uniformity of Li^+^ transport in the additive‐polymer interface by reducing the Li^+^ hopping between the additives. According to this theory, adding 2D additives to SPE should be more beneficial for Li^+^ transport because that changes from line‐to‐line transport to plane‐to‐plane transport. Luo and coworkers showed that the vertically aligned 2D vermiculite SPE exhibited Li^+^ conductivity of 1.89 × 10^−4^ S cm^−1^ at room temperature.^[^ ^155^ ^]^ The LiFePO_4_/SPE/Li cells delivered a stable capacity of 167 mAh g^−1^ at 0.1 C within 200 cycles.

Another type of SPEs is designed to immerse existing polymer/Li salt, which shows strong ion conductivity but poor mechanical properties, into ultrathin porous membranes with vertical porous channels (termed it as porous film‐based SPEs) (Figure 6h). This type of SPEs not only has a very short Li^+^ transport distance, but also has very good mechanical properties, and is one of the easiest SPEs to prepare on a large scale.^[^ ^156^ ^]^ In 2018, Cui and coworkers immersed PEO/LiTFSI into 8.6‐µm‐thick polyimide film to form an ultrathin and flexible SPE with a high Li^+^ conductivity (2.3  ×  10^−4^ S cm^−1^ at 30 °C) The LiFePO_4_/SPE/Li cells delivered a stable capacity of 105 mAh g^−1^ within 200 cycles.^[^ ^157^ ^]^

#### 5.5.2 Active Additives

Active additives introduced into SPEs can conduct Li^+^ and assist Li^+^ transport. Dispersion of small amounts of active additives into PAN‐based SPE did not affect PAN crystallinity significantly but resulted in higher Li^+^ conductivity.^[^ ^84^ ^]^ Thus, when active additives are incorporated into the SPE matrix, there is no increase ionic conductivity because the crystallinity degree decreases. Li_7_La_3_Zr_2_O_12_ (LLTO) is an acid‐site deficient perovskite‐type Li^+^ conductor. The vacancies in this compound concentrate on the surface, allowing Li^+^ to hop from one vacancy to another one.^[^ ^158^, ^159^, ^160^ ^]^ Some active additives enhance Li salt dissociation and Li^+^ liberation, increasing both Li^+^ mobility and concentration in SPEs.

**Figure 7:**
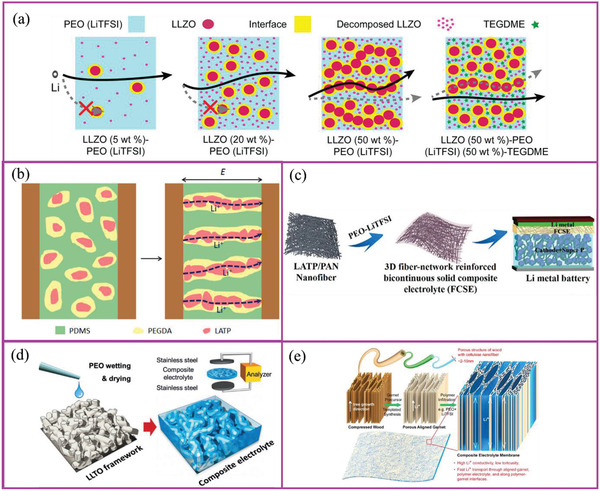
a) Li^+^ transport in SPE containing different amounts of nanoadditives. Reproduced with permission.^[^ ^161^ ^]^ Copyright 2018, American Chemical Society. Schematics showing SPEs filled with b) vertically aligned nanoparticles, c) 3D additive network, d) 3D additive framework, and e) vertically aligned active additive. Figures of (b), (c), and (e) reproduced with permission.^[^ ^251^, ^252^ ^]^ Copyright 2018, American Chemical Society. Figures of (d) reproduced with permission^[^ ^253^ ^]^ Copyright 2018, Wiley‐VCH.

Li^+^ transport always tends to occur in a uniform region of SPEs. Additive amounts in SPEs need to be optimized: insufficient additive will not improve Li^+^ transport significantly while an excess will hinder it. For example, when the LLZO concentration in PEO was in the range of 5–20 wt%, the main Li^+^ transport pathway was located inside the polymer matrix itself, and the additives played a secondary role in assisting Li^+^ transport.^[^ ^161^ ^]^ Above 20 wt%, a LLZO network formed in the polymer matrix, which blocked Li^+^ transport through it. In this case, the primary ionic transport paths were through this LLZO network. When 50 wt% of LLZO and 50 wt% of plasticizers were added, they significantly increased Li^+^ transport through the polymer matrix but not through the LLZO (**Figure** **7**a). Another research group showed that SPE conductivity increased when up to 52.5% of LLZO by weight was added.^[^ ^162^ ^]^ The addition of high amounts of LLZO resulted in a decrease in Li^+^ conductivity.

In 2018, Liu et al. demonstrated that aligned nanoparticles in SPEs can reduce the usage of nanoparticles while significantly increasing the Li^+^ conductivity.^[^ ^163^, ^164^ ^]^ Adding 4% Li_1.3_Al_0.3_Ti_1.7_(PO_4_)_3_ to poly(ethylene glycol) diacrylate and poly(dimethylsiloxane) SPE produces a Li^+^ conductivity of 2.4 × 10^−6^ S cm^−1^ without Li salt addition. This further reinforces our belief that active additives introduced into SPEs can conduct Li^+^ and assist Li^+^ transport. Therefore, there is now a greater understanding of the phase continuity of active additives in SPEs with active additive‐based 3D structures being built around polymer/Li salt complexes.

The additive phase in SPEs with a high tortuosity enhances the Li^+^ transport distance. Therefore, researchers have hypothesized that the phase continuity of active additives in SPE supports Li^+^ transport. Cui and coworkers synthesized LLTO nanowires and dispersed them in PAN‐based SPEs.^[^ ^149^ ^]^ At high LLTO nanowire concentrations, a 3D Li^+^‐conducting network formed, allowing continuous and uninterrupted Li^+^ transport (unlike situations where Li^+^ hops from one isolated particle to another). The room temperature conductivity of the resulting PAN SPE containing 15 wt% was 2.4 × 10^−4^ S cm^−1^. Randomly oriented LLTO nanowires cross‐linked with the SPE matrix, which allowed Li^+^ to move without encountering too many poorly connected interfaces.^[^ ^165^ ^]^ However, the randomness of this 3D network significantly prolonged the Li^+^ path. It has been reported recently that SPEs containing aligned LLTO nanowires oriented perpendicular to the electrodes improved Li^+^ conductivity by one order of magnitude, due to the shorter and more targeted transport paths. The Li^+^ conductivity of SPEs before and after LLTO incorporation was 3.62 × 10^−7^ and 1.02 × 10^−6^ S cm^−1^, respectively. Incorporation of randomly dispersed and aligned LLTO nanowires increased the conductivity to 5.40 × 10^−6^ and 6.05 × 10^−5^ S cm^−1^, respectively.^[^ ^84^ ^]^

For the active additive‐based 3D structures, electrospun fibers of Li_1.4_Al_0.4_Ti_1.6_(PO_4_)_3_ and Li_7_La_3_Zr_2_O_12_ are considered as an ideal continuous network with excellent mechanical strength and improved ionic conductivity for robust SPEs. Fan and coworkers showed that Li_1.4_Al_0.4_Ti_1.6_(PO_4_)_3_/PEO/LiTFSI increases its conductivity from ≈10^−6^ to ≈10^−4^ S cm^−1^.^[^ ^166^ ^]^ Yu and coworkers showed that a hydrogel‐derived Li_0.35_La_0.55_TiO_3_ framework‐based SPE can increase its conductivity to 8.8 × 10^−5^ S cm^−1^ at room temperature.^[^ ^167^ ^]^

Compared to an additive network or framework with high tortuosity, the vertically aligned structure can further shorten Li^+^ transport distance. Hu and coworkers reported that the garnet PEO/LiTFSI composite exhibited a Li^+^ conductivity of >10^−4^ S cm^−1^ at 30 °C.^[^ ^168^ ^]^ Similar results were obtained by Yang and coworkers, who used the ice‐templating‐based method to connect Li_1+_ *_x_*Al*_x_*Ti_2−_ *_x_*(PO_4_)_3_ nanoparticles to the vertically aligned nanowire.^[^ ^169^ ^]^ Such configuration retained SPE flexibility and increased its conductivity to 0.52 × 10^−4^ S cm^−1^, which was 3.6 times higher than when the same nanoparticles were only randomly dispersed.

**Figure 8:**
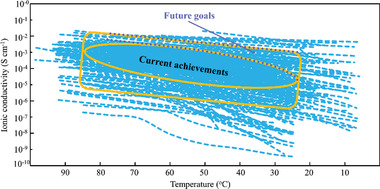
Summary of Li^+^ conductivity as a function of the temperature. The data were collected from 250 papers. The yellow‐outlined oval in the middle of the graph represents the most frequently reported conductivity values. The yellow‐outlined square represents the conductivity zone not frequently reproduced by other scientists.

To understand the current achievements fully and to provide perspectives on possible future achievements relating to Li^+^ transport in SPEs, we summarized data from 250 papers (**Figure** **8**). The majority of studies reported achieving Li^+^ conductivity in the range of ≈10^−4^ S cm^−1^ at 25–30 °C and even higher (≈10^−3^ S cm^−1^) at 50 °C. Therefore, we believe that the Li^+^ conductivity in SPEs can be above 10^−4^–10^−3^ S cm^−1^ at 30–50 °C. To develop batteries capable of operating in all weather conditions (including temperatures below freezing), additional research efforts is needed.

## 6 Strategies to Improve Li^+^ Transference Number

**Figure 9:**
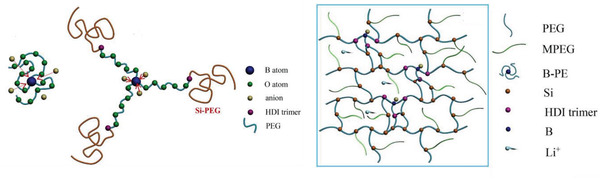
Schematic presentation of the interaction between B‐based Lewis acids and anions, resulting in LITN increase. Reproduced with permission^[^ ^177^ ^]^ Copyright 2017, Elsevier.

McCloskey and coworkers showed that LITNs above 0.7 would significantly improve battery performance.^[^ ^57^ ^]^ LITNs of current commercial electrolytes are below 0.3, which substantially limits the development of high‐performance batteries with high power density, thick electrodes, etc. Currently, the LITN of the most studied SPEs is typically ≈0.4, which is also inadequate.^[^ ^170^, ^171^ ^]^ As a result, improvements in LITN are highly sought after. Theoretically, lower LITNs of SPEs imply a faster anionic transfer than cationic transfer. For example, EO groups of PEO interact rather strongly with Li^+^, causing their transport to slow down relative to their counter anions.^[^ ^93^, ^172^ ^]^ As a result, a battery containing such SPE would exhibit polarization. Therefore, restricting anion movements or improving Li^+^ mobility are the primary ways to increase the LITNs of SPEs. Tominaga and coworkers demonstrated that Li salt concentration and polymer structure have a significant influence on LITN of SPEs.^[^ ^173^, ^174^, ^175^ ^]^ For example, more than 50 mol% lithium bis(fluorosulfonyl)imide (LiFSI) in PEC gives a high LITN of up to 0.6. This is because FSI anions tend to form aggregates, which have strong bonding with the C=O group in PEC. The LITNs of SPEs can be further improved by adding suitable additives. Tominaga et al. showed that when adding TiO_2_ to PEC/LiFSI SPE, the LITN can increase to more than 0.8 without affecting to ionic conductivity.^[^ ^176^ ^]^ Lewis acid groups and anions can be introduced into SPEs to enhance Li^+^ movement and to slow down anion movement. For example, the incorporation of borane atoms into the PEO chains traps anions and reduces the dissociation energy of Li salt; the anions are likely anchored by B atoms, thus leaving freer Li^+^. Also, having the Si‐PEG as the chain end is not only helpful in cross‐linking but also facilitates exposure of B atoms to anions. Both cases result in increased LITN and Li^+^ transport. Using this strategy, the Li^+^ conductivity and LITN were increased to 1.6 × 10^−4^ S cm^−1^ and 0.68 at 25 °C, respectively (**Figure** **9**).^[^ ^177^ ^]^

The improvement of LITN can also be achieved by enhancing the polymer chain flexibility and reducing its interaction force with Li^+^. For example, Fan and coworkers reported increased room temperature Li^+^ conductivity and LITN of SPEs up to 6.02 × 10^−3^ S cm^−1^ and 0.675, respectively, by adding 5 wt% of Al_2_O_3_ to enhance the Li^+^ dissociation energy.^[^ ^178^ ^]^ Kawakami and coworkers showed that cross‐linking a short‐chain PEO through appropriate chemical groups can significantly enhance Li^+^ movement.^[^ ^179^ ^]^ In this case, the large‐sized anions slowed down through the cross‐linked network, resulting in an increase of the LITN to 0.56.

**Figure 10:**
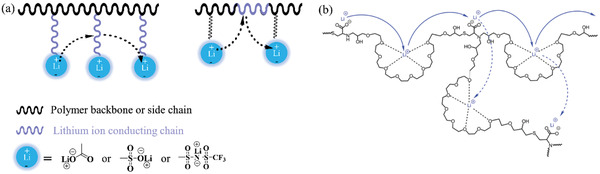
Schematic illustration of a) Li^+^ transport in single‐ion conducting polymer electrolyte and b) a representative example. Reproduced with permission.^[^ ^180^ ^]^ Copyright 2014, The Royal Society of Chemistry.

Single‐ion conducting polymer electrolytes, containing the Li source directly on the polymer chain instead of from dissolving Li salts, exhibit high LITNs. A typical structure of a single‐ion polymer includes (1) a polymer backbone, (2) ion‐transporting chains, and (3) Li^+^‐binding anionic centers.^[^ ^60^ ^]^ The main chain determines the overall mechanical properties of these SPEs, while Li^+^‐conducting side chains and the anionic center are responsible for Li^+^ transport. The Li‐polymer salt does not transport Li^+^ by itself (**Figure** **10**); it is only a Li^+^ source and can transfer Li^+^ from itself to the Li^+^ conducting chains. Single‐ion polymers are divided into two categories. The first includes polymers containing Li^+^ suspended on the conducting segments of the main chain; the second includes the conducting segments copolymerized with the main chain.^[^ ^180^ ^]^ Since the anionic center is attached to the polymer side chain and does not move, the LITNs of these single‐ion SPEs are very high.^[^ ^171^ ^]^ Anionic centers are the Li^+^ transfer points, and their binding energy with Li^+^ determines their ionic conductivity. A list of promising and well‐studied anionic centers includes carboxylates, sulfonates, and sulfonylimide anions.^[^ ^180^, ^181^, ^182^, ^183^, ^184^ ^]^ The latter anion possesses low Li^+^ dissociation energy and has been the focus of research over recent years.^[^ ^183^ ^]^

**Figure 11:**
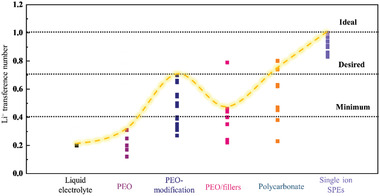
Summary of LITNs reported in the literature for different SPEs.

The LITNs of common types of SPEs are shown in (**Figure** **11**). The LITN of PEO‐based SPEs is typically slightly higher than that of liquid electrolytes, but the average value is still below 0.4. After PEO structure modification or particle addition, its LITNs increases above 0.4 and even close to 0.7. The LITN range for carbonate SPEs is wider than for modified PEO. The LITN of single ion SPEs is almost perfect, being 0.8. In this summary, we deliberately omitted some literature data. For example, the LITN of the PEO‐LiBF_4_ system is 0.81. However, the high dissociation energy of LiBF_4_ results in poor room temperature Li^+^ conductivity (only ≈10^−7^ S cm^−1^).^[^ ^185^ ^]^ Thus, to increase the LITN of a polymer, a Li salt system, different Li salts or inorganic additives are required. Thus, the most feasible way to increase LITN is to change the polymer structure of the SPE.

## 7 SPEs with Good Mechanical Properties

SPEs need to have appropriate mechanical properties in order to improve their processing, and their physical, chemical and electrochemical compatibility with cathode and anode electrodes. Newman and coworkers and Srinivasan and coworkers pointed out that SPEs with adequate shear modulus and toughness can reduce the current density, and thus inhibit the growth of lithium dendrites.^[^ ^186^, ^187^ ^]^ Balsara et al. further pointed out that a good SPE needs to have both a high ionic conductivity (>10^−4^ S cm^−1^) and a high shear modulus (*G*′ > 0.1 GPa). However, when used to increase the ionic conductivity of pure SPEs, polymer chains are generally very flexible, resulting in Li^+^ conducting group‐based SPEs exhibiting poor mechanical strength. Therefore, for Li^+^ conducting group‐based SPEs, there exists a tradeoff between Li^+^ conductivity and mechanical strength.

**Figure 12:**
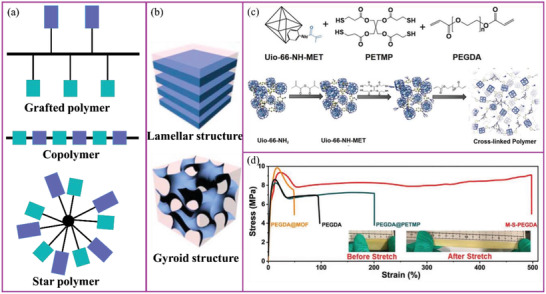
Schematic illustration of a) representative polymer structures and b) SPE morphologies. Reproduced with permission.^[^ ^81^ ^]^ Copyright 2017, American Chemical Society. c) Chemical precursors for the synthesis of cross‐linked polymers. d) Stress–strain curves of membranes from (b). Reproduced with permission.^[^ ^199^ ^]^ Copyright 2020, Wiley‐VCH.

To improve the mechanical properties of SPEs, there are four design strategies for using polymer chains. The first three methods involve the inclusion of stiff groups into the polymer by using either a copolymer, grafted polymer, or star polymer. The mechanical properties of the polymer are controlled by forming lamellar or gyroid structures, where the stiff groups are assembled into a robust mechanical phase (**Figure** **12**a,b). Balsara and coworkers systematically investigated a polystyrene‐block‐poly(ethylene oxide)‐based SPE from the aspects of molecular weight, phase separation, salt concentration, and compatibility with Li metal anode.^[^ ^103^, ^188^, ^189^, ^190^, ^191^ ^]^ At present, these kinds of polymers can increase the shear modulus by up to 0.001 Gpa.^[^ ^192^ ^]^ In addition, the star polymer formed by PEG using a stiff polymer at its core or spread in a star shape together with stiff polymer chains, also has very good ionic conductivity and mechanical properties.^[^ ^81^ ^]^ Anastasiadis and coworkers showed that the star polymer‐based SPEs can show improved shear modulus and ionic conductivity of *G*′ ≈ 0.1 GPa and 10^−3^ S cm^−1^, respectively.^[^ ^193^ ^]^ The mechanical strength can also be effectively improved by chemical crosslinking. Using appropriate chemical unit design, cross‐linked polymers can handle high intensity stress while enhancing the toughness of the polymer. Archer and coworkers and Xue and coworkers demonstrated that cross‐linked SPEs with toughness of ≈100% and ionic conductivity up to 10^−4^ S cm^−1^ can significantly improve the Li metal cycling stability.^[^ ^192^, ^194^ ^]^

Archer and coworkers created organic‐inorganic hybrid SPEs, which greatly improved the shear modulus of SPE.^[^ ^195^, ^196^, ^197^, ^198^ ^]^ The original design linked PEG with SiO_2_ nanoparticles through covalent bonds, and directly mixed them with Li salt. This type of SPE directly wraps the nanoparticles in the PEG core, so the ion‐conducting polymer phase has continuity and does not affect ion transport. It is worth noting that nanoparticles are uniformly dispersed in this type of SPE, and they will change from a jammed to an unjammed state during the deformation process, and be reversible. A recent study by Archer et al. has shown that chemical cross‐linking of organic hybrid SPEs can increase the shear modulus by an order of magnitude and further improve its stability to a Li metal anode.^[^ ^113^ ^]^ Sun and coworkers designed a cross‐linked SPE with excellent mechanical strength (9.4 MPa)/toughness (≈500%) and high Li^+^ conductivity (2.26 × 10^−4^ S cm^−1^ at room temperature) (Figure 12c,d).^[^ ^199^ ^]^ By uniformly introducing MOFs (UIO‐66) particles into the tetrakis(3‐mercaptopropionic acid) pentaerythritol (PETMP) and poly(ethylene glycol) diacrylate (PEGDA) network, the mechanical strength is the same as the PETMP‐PEGDA network but the toughness is increased more than 2.5 times. As a result, the cell of LiFePO_4_/SPE/Li can deliver a stable capacity of 123.1 mAh g^−1^ at 0.5 C for 500 cycles.

The fourth method to improve the mechanical properties of SPE is to immerse a polymer/Li salt directly into a framework with good mechanical properties. The prepared composite SPE then has mechanical properties similar to the skeleton.^[^ ^200^ ^]^ Tominaga and coworkers used polyimide as a three‐dimensional structure, allowing PEC/LiFSI SPE to form a self‐supporting membrane.^[^ ^175^ ^]^ When Fan and coworkers combined poly(vinylidene fluoride)) (PVDF) polymer/LiTFSI immersed into the electrospinning framework, the tensile strength of the SPE increased from 3.9 to 11.5 MPa.^[^ ^201^ ^]^ The Capacity retention of LiNi_0.5_Co_0.2_Mn_0.3_O_2_/SPE/Li increased from 75% to 95% after 80 cycles.

## 8 Typical Applications of SPEs

### 8.1 High Energy Systems

The replacement of current commercial electrolytes with SPEs will not significantly improve the performance of existing battery systems. However, if the SPEs were paired with Li metal anodes and high‐voltage/high‐capacity cathode materials, the energy density of the resulting battery would increase dramatically.^[^ ^15^, ^202^, ^203^, ^204^, ^205^ ^]^ However, the main focus of such a battery still needs to be on its safety and stability.^[^ ^70^, ^206^ ^]^ High‐voltage or high‐capacity cathode materials are always accompanied by exothermic interfacial reactions.^[^ ^207^, ^208^, ^209^ ^]^ Uneven ion transport at the Li metal anode interface can cause electrolyte polarization and interfacial instability, which are also the main reasons for capacity decay and compromised battery safety. Thus, this section focuses on the achievements in SPE development dedicated to suppressing Li dendrite formation. This section also describes SPEs that have been explicitly developed for high energy density batteries.

**Figure 13:**
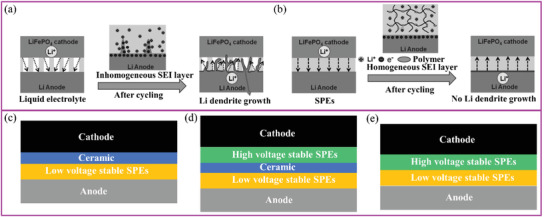
Li^+^ deposition in a) liquid electrolyte and in b) SPEs. Reproduced with permission.^[^ ^211^ ^]^ Copyright 2017, Wiley‐VCH. c–e) Schematics showing how the incorporation of different layers (low and high voltage SPEs and ceramic layers) can improve battery performance and expand electrochemical operation windows of SPEs.

Li metal is considered the best anode material for LBRBs because of its highest theoretical specific capacity (≈3860 mAh g^−1^) and lowest redox potential (−3.04 V vs standard hydrogen electrode).^[^ ^210^ ^]^ However, its unstable interface reacts readily with the electrolyte. This reaction generates gas and heat, which often leads to battery decay. In addition, the uneven deposition of Li^+^ often causes Li dendrite growth, which is, by itself, a serious safety problem (**Figure** **13**a,b).^[^ ^211^ ^]^ Thus, more uniform Li^+^ conduction at the interface is needed, which can be achieved by optimizing Li^+^ transport at the SPE interface and inside the SPE itself.^[^ ^15^ ^]^ The ideal ways to achieve this (through reduction of SPE crystallinity degree and/or additive addition) have already been discussed in the previous sections.

The poor wettability at the SPE/Li metal interface (especially when compared with that of the liquid electrolyte) creates a favorable environment for Li dendrite growth in the SPE/electrode gap. Therefore, modification of the interface between SPEs and electrodes should improve ion transport.^[^ ^209^ ^]^ Archer and coworkers polymerized 1,3‐dioxolane in situ onto the Li metal surface to obtain an SPE with high room temperature Li^+^ conductivity (>1 mS cm^−1^).^[^ ^212^ ^]^ Other advantages of this design were low interfacial resistance and uniform Li plating/stripping efficiency. The Coulombic efficiency of the cells assembled using this SPE, with Li metal as an anode, and LiFePO_4_ as a cathode active material, was 100% even after 700th cycles.

Goodenough and coworkers stated that “no single polymer or liquid electrolyte has a large enough energy gap between the empty and occupied electronic states for both dendrite‐free plating of a lithium‐metal anode and a Li^+^ extraction from an oxide host cathode without electrolyte oxidation in a high‐voltage cell during the charge process.”^[^ ^213^ ^]^ High‐voltage cells require additional materials to either decompose or protect the electrode interfaces in order to inhibit side reactions. Three different designs of batteries containing SPEs are shown in Figure 13c–e.^[^ ^124^, ^213^, ^214^, ^215^ ^]^ Incorporation of a ceramic layer is beneficial because it protects the battery from a high‐voltage state and can provide higher LITNs. However, interface compatibility between the ceramic layers, the rest of the active materials and SPEs is often poor, which results in impedance increase. Typically, ceramic layers are placed on the cathode side, or between the two polymer layers (Figure 13c,d). SPEs next to the Li metal are needed to have excellent mechanical properties, flexibility, and uniform Li^+^ transport to suppress dendrite growth.^[^ ^215^ ^]^ SPEs, stable at high voltages, can also be used for the cathode to improve the interface compatibility. A battery assembled using an electrolyte consisting of polymer/ceramic membrane/polymer electrolyte layers, Li metal as the anode, and LiFePO_4_ as a cathode active material, showed excellent cycling performance with 99.8−100% Coulombic efficiency over 640 cycles at 0.6 C.^[^ ^124^ ^]^ A combination of SPEs that are stable at high and low voltages can simultaneously protect both cathode and anode, respectively. Goodenough and coworkers showed that a PEO layer placed on the Li metal side and poly(*N*‐methyl‐malonic amide) placed on the cathode side stabilized the Li/LiCoO_2_ cell for over 100 cycles at voltage exceeding 4.2 V (Figure 13e).^[^ ^213^ ^]^ Incorporation of different SPE layers can also be used to improve the performance of flexible batteries. It is strongly believed that this is the next direction of development of high energy density batteries. Both traditional SPEs and liquid electrolytes operate by providing anion and cation transport during battery charging and discharging. During this process, high Li concentration gradients form in the battery system, which often leads to uneven Li^+^ deposition on the Li metal surface.^[^ ^216^ ^]^ Implementation of single‐ion polymer electrolytes in batteries can effectively reduce this concentration gradient and inhibit the Li dendrite formation.^[^ ^60^ ^]^ For example, use of multiblock co‐poly(arylene ether sulfone) membranes with a LITN equal to 1 and high Li^+^ conductivity (equal to ≈10^−3^ S cm^−1^ above 30 °C) showed stable Li^+^ stripping/plating cycling for 800 h at 0.5 mA.^[^ ^217^ ^]^ Feng and coworkers prepared a single‐ion SPE with maleic anhydride and lithium 4‐styrenesulfonyl(phenylsulfonyl)imide. The room temperature LITN and ionic conductivity of this composite SPE were equal to 0.97 and 3.08 × 10^−4^ S cm^−1^, respectively. The assembled Li/LFP cell kept about 90% of its initial capacity after 350 cycles at 0.1 C.^[^ ^218^ ^]^

It is equally important to improve the compatibility of cathode and SPEs, and increase the stability of SPEs or the interface of cathode materials. For example, Tominaga and coworkers demonstrated that SPEs with a high Li salt concentration can improve the electrochemical window up to 5.5 V and protect current collector corrosion.^[^ ^219^ ^]^ Yang and coworkers reported that a solid inorganic electrolyte coated LiCoO_2_ cell can improve it compatibility with PEO‐based SPE at 4.25 V.^[^ ^220^ ^]^ The cycling performance of a Li/SPE/LiCoO_2_ cell can increase from 100 cycles to 400 cycles with a capacity retention of 81.9%.

### 8.2 Safe Devices

**Figure 14:**
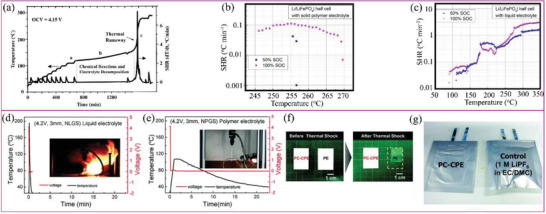
a) Temperature changes during accelerating rate calorimetry (ARC). Reproduced with permission.^[^ ^30^ ^]^ Copyright 1999, The Electrochemical Society, Inc. Self‐heating rate (SHR) of the batteries containing b) SPEs and c) liquid electrolytes obtained using ARC. Reproduced with permission.^[^ ^225^ ^]^ Copyright 2016, Elsevier. Temperature and voltage changes of the batteries containing d) liquid electrolytes and e) SPE during the nail penetration process. Reproduced with permission.^[^ ^226^ ^]^ Copyright 2015, The Royal Society of Chemistry. f) Changes of SPE and PE separator, as well as of the pouch cells g) containing SPE (PC‐CPE) and PE (Control) after thermal shock. Reproduced with permission.^[^ ^32^ ^]^ Copyright 2016, The Royal Society of Chemistry.

The interface reactions between the electrolyte and the electrode, as well as electrolyte flammability and thermal shrinkage of the separator, greatly contribute to the safety issues around Li‐based batteries.^[^ ^221^, ^222^, ^223^ ^]^ The decomposition of liquid electrolyte on the cathode is one of the main reasons for battery thermal runaway (**Figure** **14**a).^[^ ^30^ ^]^ When the battery is subjected to abuse conditions or experiences a mechanical shock, the electrolyte is the main factor determining what happens next. Currently, commercialized liquid electrolytes are combustible and volatile.^[^ ^221^ ^]^ In addition, internal short circuits caused by separator damage are the root cause of battery accidents.^[^ ^222^ ^]^ Widely‐used commercial Celgard separator material shrinks and melts at 140–160 °C, causing a short circuit in the battery and rapid heat generation, both of which directly lead to battery thermal runaway.^[^ ^224^ ^]^ Polymer‐based SPEs react slowly or do not at all, with the electrode materials, improving heat diffusion and thus suppressing thermal runaway (Figure 14b,c). A fully‐charged battery containing liquid electrolyte starts to generate heat at 90 °C due to the decomposition of the electrolyte on the anode side (Figure 14c). Liquid electrolyte decomposition at the cathode interface continues to generate heat, which causes cathode material to start releasing oxygen, which, in turn, reacts with other battery materials.^[^ ^225^ ^]^ These processes sharply increase the battery temperature. The stability of SPEs ensures that the assembled battery will only react at temperatures over 247 °C. Thus, the reactions and the SPE/electrode interface will not contribute to battery thermal runaway. As the accelerating rate calorimeter (ARC) data in Figure 14b,c show, the maximum self‐heating rate (SHR) of a cell containing liquid electrolyte is 3.2 °C min^−1^, while the maximum SHR of an SPE‐based battery is only 0.11 °C min^−1^ (Figure 14b). Nail penetration tests, widely used to test battery stability, carried out on pouch cells containing traditional liquid electrolyte resulted in the appearance of smoke, followed by violent burning with the cell temperature reaching over 200 °C after just a few seconds.^[^ ^226^ ^]^ However, the SPE structure remained unchanged during nail penetration. The maximum surface temperature of the SPE‐containing cell was only 105 °C, and the heat and gas generation were much lower than that from the batteries containing liquid electrolytes (Figure 14d,e).

**Figure 15:**
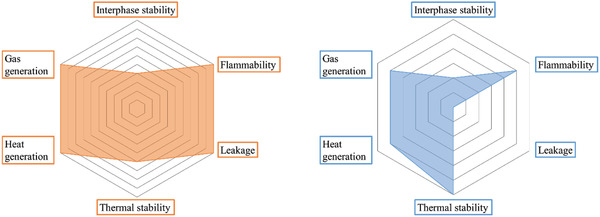
Radar plots comparing interphase stability, flammability, leakage, thermal stability, and heat and gas generation of liquid electrolyte (right) and SPEs (left).

Another advantage of SPEs is their solid form. Thus, in the case of mechanical damage, there is no electrolyte leakage, and side reactions with the electrodes will be limited due to slow diffusion between solids. Guo and coworkers cut SPE‐based batteries and the battery continued to operate normally, with no voltage changes or accidents (e.g., fire) occurring.^[^ ^214^ ^]^ Separator damage caused by elevated temperatures is a serious issue as well.^[^ ^227^, ^228^ ^]^ Optimization of polymer substrate and inorganic additives is helpful in maximizing the SPE thermal shock tolerance. For example, Lee and coworkers demonstrated that under the same thermal shock conditions, polyethylene (PE) separator shrank by 43%, while the shrinkage of the SPEs was negligible (Figure 14f).^[^ ^32^ ^]^ After the thermal shock, the battery with the PE separator no longer worked and swelled severely (Figure 14g). Electrolyte burning is another severe problem during battery thermal runaway. The burning of SPEs is generally limited due to a lower C:H ratio and air‐contact area in comparison to liquid electrolytes. In addition, diverse polymer structures can be used for SPE backbone, and flame‐retarding groups (such as groups with high carbon content, phospholipids, halogens, etc.) can be introduced into the SPEs to improve their fire‐retarding property.^[^ ^229^, ^230^ ^]^ As heat and gas from the battery are the main reasons for fire and explosions, SPEs are very promising components for battery safety improvements with their excellent interfacial inertness, low flammability, no leakage, and high thermal stability. Incorporation of SPEs into batteries can avoid gas volatilization and will stabilize the material interface, significantly reducing battery heat and gas generation (**Figure** **15**).

### 8.3 Flexible Batteries

**Figure 16:**
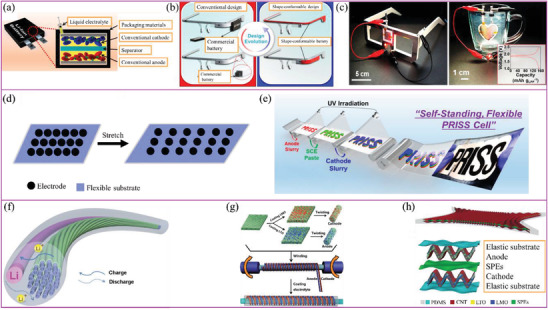
Schematics showing the internal structure of a) traditional batteries and b) differences in manufacturing process between the traditional and flexible batteries. c) Fabrication of flexible batteries with flexible structures at a laboratory scale. Reproduced with permission.^[^ ^231^ ^]^ Copyright 2017, American Chemical Society. d) Batteries based on flexible substrates. e) Printable solid‐state LBRBs. Reproduced with permission.^[^ ^231^ ^]^ Copyright 2017, American Chemical Society. f) Wire‐shaped LBRBs. Reproduced with permission.^[^ ^233^ ^]^ Copyright 2017, Wiley‐VCH. g) Stretchable wire‐shaped LBRBs. Reproduced with permission.^[^ ^234^ ^]^ Copyright 2017, The Royal Society of Chemistry. h) Wavy stretchable LBRBs. Reproduced with permission.^[^ ^236^ ^]^ Copyright 2018, Wiley‐VCH.

A flexible battery needs flexible components.^[^ ^35^ ^]^ SPEs are currently the ideal electrolytes to use in high‐performance flexible batteries, because their flexible polymer backbones are able to accommodate reversible stretching and bending without failures.^[^ ^34^ ^]^ Although the traditional battery and the flexible battery have similar working principles, traditional battery architectures contain liquid electrolytes and almost unstretchable separators (**Figure** **16**a).^[^ ^231^ ^]^ When deformed, the electrolyte might become unevenly distributed/squeezed, and the separator might not recover its original shape. Both of these factors might adversely affect battery performance and safety. A traditional battery has a predetermined size allowing it to be used in specific devices. The size of a flexible battery can be variable, which can be realized by designing specific thin layers of various shapes (Figure 16b,c). Thus, if performance issues of the flexible batteries can be overcome, flexible electronic devices (e.g., wearable, electronic skin, etc.) can be manufactured using SPEs.^[^ ^231^ ^]^

This review focused on four types of flexible batteries. The first type is based on the cathode and anode materials currently used for LBRBs but built with flexible SPEs and substrates. Building block design is used to achieve the required flexibility, where small and rigid cells are building blocks connected with deformable joints. Rogers and coworkers fabricated a flexible battery by positioning multiple Li_4_Ti_5_O_12_ anodes (1.58 mm in diameter) and LiCoO_2_ cathodes (2.20 mm in diameter) on opposite sides of an SPE layer. After a 300% stretch and 20 cycles, the battery capacity stayed at 1.1 mAh cm^−2^ (Figure 16d).^[^ ^232^ ^]^ The area capacity of the flexible battery is ≈50% that of traditional batteries. Such flexible batteries can be used for stretchable and bendable devices. The second type of flexible battery is a solid‐state battery, where the sandwich structure is ink‐jet printed layer by layer. Such printable solid‐state batteries could be integrated with wearable eyeglasses (Figure 16c) as printing parameters define the battery shape and can be very diverse (Figure 16e).^[^ ^231^ ^]^ Such flexible batteries are suitable for complex and multidimensional/multiscale devices (Figure 16c,e). The third type contains flexible wire‐shaped LBRBs, which are typically fabricated using highly conductive fibrous cathode and anode materials filled with SPEs (Figure 16f).^[^ ^233^ ^]^ Such a design eliminates the usage of rigid materials such as current collectors. Peng and coworkers assembled batteries using MWCNT/LiMn_2_O_4_ composite fibers as a cathode and MWCNT/Li_4_Ti_5_O_12_ composite as an anode, respectively (Figure 16g).^[^ ^234^ ^]^ The resulting battery could be stretched up to 600% while maintaining 88% of its initial capacity. This type of battery can be woven into textiles to fabricate smart clothing or be directly integrated with flexible circuits, computer interfaces, etc.^[^ ^235^ ^]^ The fourth type is flexible and stretchable LBRBs with wavy electrodes. These batteries contain stretchable SPEs. For example, Peng and coworkers mixed LiMn_2_O_4_ and Li_4_Ti_5_O_12_ with rippled CNT sheets and sandwiched the electrodes with SPE. The resulting battery could tolerate 500 cycles of up to 400% of stretching deformation (Figure 16h).^[^ ^236^ ^]^ Based on flexible batteries, another field of SPE applications emerging is transparent batteries and devices (such as touch and display screens).^[^ ^237^, ^238^, ^239^ ^]^ Scientific research on transparent SPEs is ongoing, and several transparent battery prototypes have already been reported in the literature.^[^ ^136^, ^240^, ^241^, ^242^, ^243^, ^244^, ^245^, ^246^, ^247^ ^]^

## 9 Conclusions and Perspectives

Over the past few decades, significant progress has been made in the field of solid polymer electrolytes. Numerous functional groups, such as imine, boryl, carbonyl, cyan, etc. have been investigated for use in SPE structural design and exhibited their effectiveness as well as promising performance. Moderately strong bonding between Li^+^ and the Lewis‐acidic polymer units, such as boron ether, facilitate Li^+^ diffusivity in polymer chains. Carbonyl‐based poly(propylene carbonate) possesses the highest room‐temperature ionic conductivity of all SPEs. Additionally, PAN is attracting a lot of research attention due to its high Li^+^ conductivity and excellent physical properties. Nevertheless, Li^+^ ion conductivity is still a barrier to SPE applications. To enhance the conductivity, the most effective way is to optimize polymer chain movement by designing superior polymer backbones. Oligomers and other active groups can be grafted onto polymer backbones to reduce overall crystallinity, while copolymers can induce regular phase separation. Additives help to enhance the ionic conductivity because the interaction of the particles with the polymer segments provides a favorable environment for Li^+^ transport in the matrix. Additives are also becoming popular since they do not affect the polymer structure but are capable of assisting Li^+^ transport. The addition of oriented additives significantly improves SPE ionic conductivity (up to 10^−4^ S cm^−1^) by shortening the transport pathway of Li^+^ ions, which is very promising for practical applications. Research on next‐generation SPEs is needed to help design highly conductive polymers by further understanding their ion transport properties, such as ion exchange between the polymer chains. The discovery and development of new active groups are critical for producing efficient SPEs, but at present, this topic is hardly covered in the literature. At the same time, there is a great deal of literature about the use of additives to modify SPEs. However, fundamental understanding of the additive‐assisted Li^+^ transport in SPEs, as well as analysis of the structure/activity relationship of the additive structure, arrangement and interphase with Li^+^ conductivity, is still lacking. It is believed that SPE will be use to produce flexible and high‐energy batteries. SPE stability including the thermal/chemical/electrochemical/mechanical stability of SPEs, the durability of SPE‐based batteries under various conditions (including bending, stretching, reshaping, etc.), and safety evaluation of SPE‐based batteries will be investigated in the near future. Advanced characterization techniques, such as neutron diffraction, small‐angle X‐ray scattering, in situ and operando tools, etc. will probably be used in these investigations.

## Conflict of Interest

The authors declare no conflict of interest.
